# Minimal invasive microscopic tooth preparation in esthetic restoration: a specialist consensus

**DOI:** 10.1038/s41368-019-0057-y

**Published:** 2019-10-02

**Authors:** Haiyang Yu, Yuwei Zhao, Junying Li, Tian Luo, Jing Gao, Hongchen Liu, Weicai Liu, Feng Liu, Ke Zhao, Fei Liu, Chufan Ma, Juergen M. Setz, Shanshan Liang, Lin Fan, Shanshan Gao, Zhuoli Zhu, Jiefei Shen, Jian Wang, Zhimin Zhu, Xuedong Zhou

**Affiliations:** 1 State Key Laboratory of Oral Diseases & National Clinical Research Center for Oral Diseases & West China Hospital of Stomatology, Sichuan University, Chengdu, China; 2 Chinese PLA General Hospital, Chinese PLA Medical Academy, Yantai, China; 3 Department of Stomatology Digitization, Hospital of Stomatology, Tongji University, Shanghai, China; 4 Department of Prosthodontics, Hospital of Stomatology, Peking University, Shanghai, China; 5 Department of Prosthodontics, Guanghua Stomatological Hospital, Sun Yat-sen University, Guangzhou, China; 6 Department of Biologic and Materials Sciences and Division of Prosthodontics, University of Michigan School of Dentistry, Ann Arbor, MI USA; 7 Department of Prosthodontics, School of Stomatology, The Fourth Military Medical University, Xi’an, China; 8 Department of Prosthodontics, Hospital of Stomatology, Martin-Luther-University, Halle (Saale), Germany; 9 Department of Prosthodontics, Hospital of Stomatology, Wuhan University, Wuhan, China

**Keywords:** Fixed prosthodontics, Dental caries

## Abstract

By removing a part of the structure, the tooth preparation provides restorative space, bonding surface, and finish line for various restorations on abutment. Preparation technique plays critical role in achieving the optimal result of tooth preparation. With successful application of microscope in endodontics for >30 years, there is a full expectation of microscopic dentistry. However, as relatively little progress has been made in the application of microscopic dentistry in prosthodontics, the following assumptions have been proposed: Is it suitable to choose the tooth preparation technique under the naked eye in the microscopic vision? Is there a more accurate preparation technology intended for the microscope? To obtain long-term stable therapeutic effects, is it much easier to achieve maximum tooth preservation and retinal protection and maintain periodontal tissue and oral function health under microscopic vision? Whether the microscopic prosthodontics is a gimmick or a breakthrough in obtaining an ideal tooth preparation should be resolved in microscopic tooth preparation. This article attempts to illustrate the concept, core elements, and indications of microscopic minimally invasive tooth preparation, physiological basis of dental pulp, periodontium and functions involved in tool preparation, position ergonomics and visual basis for dentists, comparison of tooth preparation by naked eyes and a microscope, and comparison of different designs of microscopic minimally invasive tooth preparation techniques. Furthermore, a clinical protocol for microscopic minimally invasive tooth preparation based on target restorative space guide plate has been put forward and new insights on the quantity and shape of microscopic minimally invasive tooth preparation has been provided.

## Introduction

Through preventive and early-stage disease treatment methods, minimally invasive dentistry has remained a conservative concept for maintaining tooth and periodontal structures with the least tooth tissue reduced or replaced.^1^ This concept has been put forward by Simonsen^2^ in 1987, and since then the citation frequency of “minimally invasive dentistry” has been rising with time. To date, about 79 600 items related to this concept were found through Google scholar. Minimally invasive dentistry is not limited to caries treatment, but its principle can also be used in any general field of dentistry.^3^ In esthetic dentistry, the concepts of minimally invasive cosmetic dentistry (MICD) and minimally invasive prosthetic procedure (MIPP) have been widely recognized. MICD involves a series of minimally invasive treatments to achieve a pleasing esthetic dental effect. The concept was first put forwarded by Sushil Koiral^4^ in the book of “*Cosmetic Dentistry - Science and Beauty*” in 2009. Comprehensively, MICD combines the functional and esthetic aspects to design minimally invasive treatments and do not compromise the esthetic, psychological, or physiology aspects of patients. MICD mainly emphasized on patients’ benefits. Conventional treatment always focuses on the objective factors of minimally invasive dentistry,^5^ while MICD brought the attention to the subjective factors of patient psychology. The concept of MIPP involves clinical procedure that focuses on the use of minimally invasive treatments to achieve a high standard of esthetic dental function and long-term success. It was brought up by Professor Mauro Fradeani,^6^ the associate editor of *European Journal of Esthetic Dentistry*. The comprehensive concept of MIPP was especially appropriate for dental attrition with reduced occlusive height, which could be reconstructed with minimal reduction of the tooth tissue.^7^ Long-term restoration outcomes reported that the principle of MIPP was suitable for esthetic rehabilitation as well.^8^ The core principle for both MICD and MIPP was maximum preservation of tooth tissue, which is also one of the earliest consensus for the preparation of dental treatments in esthetic area.^9^

Currently, the regular method used for esthetic restorations is to remove certain amount of tooth tissue and replace the space with ceramic restorations.^10^ Porcelain esthetic restorations include porcelain veneers, porcelain crowns, porcelain inlays, etc. Ceramic veneers have superior properties in both esthetic and tooth preservations and are considered as minimally invasive treatment for indirect esthetic restoration.^11^ The development of dental ceramic techniques offered a veneer thickness of about 0.3–0.5 mm, decreasing tooth reduction amount and ensuring it to be within the enamel structure and effective bonding.^12^ Without the exposure of dentin, sensitivity discomfort would be alleviated and enamel bonding interface also demonstrated to have higher strength. Nevertheless, studies have shown excessive invasion for some of the veneer tooth preparations.^13^ Practically, dentin exposure occurs when the prepared finishing planes exceeded the enamel–dentin junction during clinical operations.^14^ Poor long-term effects from non-minimally invasive operations occur due to decreased bonding strength, increased dentin sensitivity, and microleakage. The unfavorable phenomena were often caused by inaccurate clinical treatment designs and imprecise tooth preparations, such as free hand tooth preparation. Group of specialists of Dental Show West China Conference met to discuss the results of the review; consensus statements and clinical recommendations arising from the review were then discussed and agreed upon, then presented to a complete session for discussion and concluding agreement.

## Consensus Statement

### Indications

The concept of minimal invasion involves possible preservation of most tooth and periodontal tissues and is the major principle involved in dentistry. In 1975, Rochette^15^ first described a minimal tooth preparation technique for anterior teeth, which made ceramic restorations for fractured incisors without operative interference. This method followed the procedure of fusion of porcelain layers onto gold foil-covered dies, restoration of surface silanization, and bonding it onto the etched tooth enamel with resin. Horn, Christensen, and Friedman^16–18^ promoted this technique in 1980s and built the foundation for minimally invasive ceramic veneers.^19^ Even though the development of bonding technique and material science guarantees a preferably long-term effect, the condition not in every case allows for the application of minimally invasive tooth preparation.^20^ Minimally invasive tooth preparation can be used when the targeted restorative space (TRS) is designed with the requirement of minimally invasive space reserve.^21^ In a word, minimally invasive tooth preparation is only a means of tooth preparation and requires minimally invasive space. Otherwise, in order to realize the patient is complaint, it is necessary to remove more tooth tissues rather than minimally invasive preparation technology.

Owing to basic conservative principle, the non-invasive techniques such as bleaching remain satisfactory, and hence tooth preparation is not required.^22^ For example, chemical methods are recommended for mild tooth discoloration (such as dental fluorosis and mottled enamel from the tooth trauma during tooth development).^23^ If the non-invasive methods could not fulfill the esthetic and mechanical goals, after the analysis of TRS, the minimal invasive or conventional tooth preparations should then be considered.

Minimal invasive preparation involves color, contour, and position changes for abutment teeth. However, owing to limited restoration space provided, the promotion remained mild. For severe discoloration, the minimal invasive results insist on compromised restoration effects. However, compared with traditional tooth preparations, the enamel preservation improves bonding properties and stabilizes the original tooth mechanics, leading to better long-term effects.^24^ Therefore, practitioners should keep in mind the indications of minimally invasive tooth preparation.^25^

Indications of microscopic minimal invasive preparation include^26–32^:

1. Mild and moderate tooth discoloration (such as tetracycline pigmentation teeth and dental fluorosis) with unsatisfying effects for tooth bleaching or denying tooth bleaching
2. Mild congenital or acquired tooth malformation (such as hypoplasia or hypocalcification of enamel)
3. Class III, IV, and V cavity restoration with unsatisfying color
4. Anterior teeth diastema and black triangle correction
5. Small defect at incisal edge
6. Malformations such as microdontia and conical teeth
7. Mild twisting or malalignment of anterior teeth, denying for orthodontics treatment
8. Mild lingual inclination of anterior teeth, denying for orthodontics treatment
9. Demands of teeth contour promotion (e.g., increased incisal length or teeth convex)

### Physiological basis of minimally invasive tooth preparation

The early-stage rehabilitation treatments often require massive tooth tissue, but the restoration material and adhesive techniques are limited. However, the tooth tissue is limited and currently not clinically reproducible, and so the accuracy of tooth preparation remains to be very important. The attention was mainly focused on avoiding stimulation and damage of tooth pulp.^33^ Owing to tooth anatomical considerations, the pulp protection is influenced by the thickness of enamel–dentin complex and remaining dentin.^10^ In most of the cases, the lingual surface shares the thinnest enamel–dentin complex (1.7–2.6 mm) in the esthetic area,^34^ and the effect is mostly evident for the maxillary lateral incisors. The full crown preparation involves the path of insertion and pulp exposure, which mostly occurs at the site of pulp horn. Currently, owing to the development of restoration material and adhesive techniques, enamel bonding showed satisfactory long-term outcomes. In minimally invasive esthetic restorations, the aim is how to control the amount of removal during tooth preparation and minimization of dentin exposure and how to avoid the impact on dental pulp tissue due to varied intraoperative and postoperative stimulations.

**Fig. 1:**
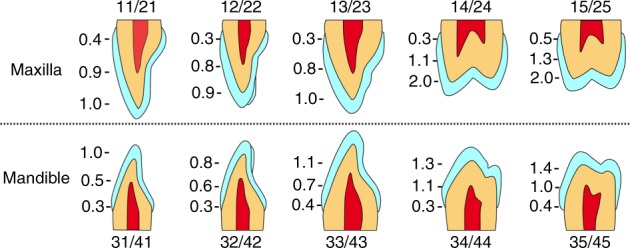
The enamel thickness in esthetic area

First, the enamel should be prepared. Previous scholars believed that a 0.5-mm hard tissue reduction from tooth is considered to be ideal for bonding space. Nattress and Cherukara^13^ then questioned that 0.5 mm reduction would cause dentin exposure in the third and proximal areas of the cervical region. Data from Ferrari’s^35^ report revealed the anatomical reason for this. By measuring the enamel thickness at the incisal third, the medium third, and the cervical third of 114 anterior teeth, Ferrari found that the average thickness of enamel at cervical third should be 0.3–0.4 mm in thickness (Fig. 1). Except for anatomical reasons, age-related attrition and tooth erosion also accelerated the attenuation of enamel. Therefore, minimal invasive tooth preparation within the enamel is quite challenging.^36^ Taking 0.5 mm porcelain veneer as an example, it has been agreed that the amount of preparation is not a fixed single value, but the value ranged from 0.3 to 0.7 mm. At present, the tooth prepared by ceramic veneer should be <0.7 mm on the incisal third, <0.5 mm for the middle third, and <0.3 mm for the cervical third.

For the protection of pulp, the Cox’s^37^ study reported that the pulp alteration occurs when the residual dentin thickness is <1 mm. Murray et al.^38,39^ further found that, when the tooth tissue is <0.5 mm for tooth preparation finishing of within 0.5 mm to the pulp cavity, the young teeth pulp may produce irreversible lesions. Pulpitis degree has been decreased with increasing thickness of residual dentine. Therefore, to obtain long-term stability, the pulp should be protected, the amount of tooth preparation should be reduced, and the cutting surface of tooth preparation within tooth enamel should be accurately controlled. This is the prevailing concept of minimally invasive tooth preparation.^40^

### Treatment designing of minimally invasive tooth preparation

The aim of tooth preparation in the esthetic zone is to achieve a sufficient and even space for future ceramic restoration. Usually, the final restoration contour does not completely overlap with the original human teeth. Therefore, tooth preparation designing should be carried out preoperatively by referring to TRS,^21^ rather than evaluating by a rough naked eye during the operation.

**Fig. 2:**
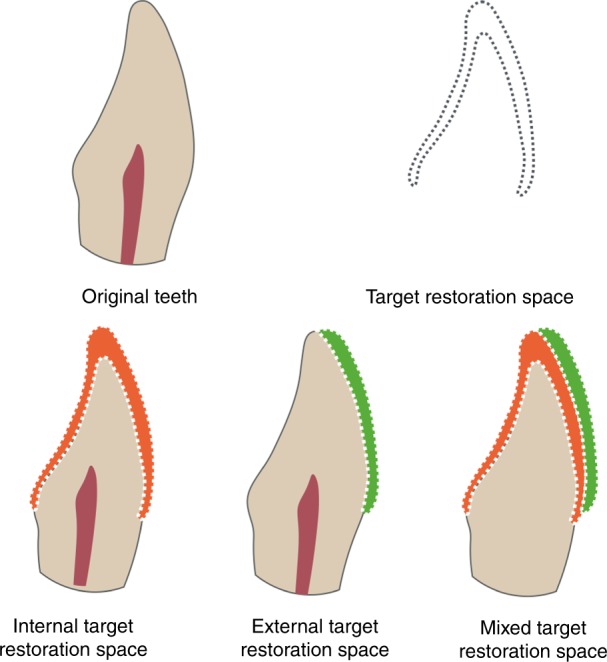
Classification of targeted restorative space

TRS stands for minimal space that is needed by the restoration to achieve the restorative treatment goals of esthetic and function. In other words, the preparation space made by the dentists in esthetic dentistry and the process of restoration therapy should meet the shape and position of the target restoration by considering the esthetic and function along with the situation of patients. Compared with the space of untreated tooth, TRS is classified into internal targeted restorative space (ITRS), external targeted restorative space (ETRS), and mixed targeted restorative space (MTRS). (1) ITRS: This TRS falls within the original tooth space. ITRS is often found in cases undergoing restoration of original contour, such as tooth crown fracture cases with intact contralateral teeth reference or veneer restoration treatments for color promotion. (2) ETRS: This TRS is completely outside the original tooth space. This kind of space is common in cases requiring expansion of teeth space based on original tooth contour, for example, during the non-invasive restorative treatment, the anterior teeth scattered space closure, and the direct noninvasive resin veneers for tooth discoloration or non-preparation of ultra-thin ceramic veneer treatments. (3)MTRS: This TRS partly overlaps with the original tooth space. The majority of esthetic cases belong to this classification, such as the twisted tooth restoration, protrusion or retraction of tooth, etc. (Fig. 2)

The esthetic TRS is often considered as a mixed space. Only the detailed TRS space design before surgery can obtain precise tool preparation amount, preserve the tooth, and protect the vital pulp, thereby achieving the goal of minimally invasive treatment.^41^

### Implementation of minimal invasive tooth preparation

Tooth preparation refers to the use of dental cutting tools (including rotary cutting tools and non-rotating cutting tools) during oral treatment to remove the tooth tissue partly and prepare for specific resistance, retention, and finish lines to provide good space and supporting structure for future restorations. Tooth preparation includes two core elements: quantity and shape. “Quantity” mainly provides suitable space for future restorations and is mainly related to the depth and scope of preparation. “Shape” mainly refers to the TRS space boundary, including the finish line, shoulder and tooth cutting surface, etc.

After designing the TRS, it is necessary to study on how to transform the designed TRS into a precise preparation amount for maximizing the preserved preparation after preparation and achieving the final restoration of the target, which remains the core of minimally invasive restoration. To achieve this goal, from a clinical implementation perspective, the operator should obtain a better view with the use of oral magnifying devices. The use of a tooth preparation guide and the corresponding tooth preparation guiding technology makes it possible to obtain a precise designed preparation amount, a maximum preparation preservation, reasonable shape of the abutment tooth, and to create the necessary space for the realization of final target restoration.^42^

However, in the era of traditional restoration by naked eyes, the restoration was often unable to control and measure the tooth preparation volume, the position and shape of the edge and the tightness cannot be finely controlled, or the operation field was not fine enough. The following complications often occurred during restoration: abutment pain, gingival recession, red and swollen, fracture, or debonding of the restoration. Restoration under the view of naked eyes cannot easily achieve the results of minimally invasive dentistry. Minimal invasive restoration can avoid the unfavorable factors that could not easily be done by traditional restoration under the naked eyes, preserve more tooth tissues to protect the vitality of dental pulp, make the effect of restoration stable and effective for a long time, and naturally achieve the results of minimal invasive restoration with respect to those under the naked eyes.

Compared to the conventional oral clinical techniques, minimal invasive restoration had the following advantages:

Better field of view

Microscope can provide 3–20 times or more of magnification, allowing the dentist to identify the fine tooth structures and perform more precise treatments. The field of view at high magnification field, such as 16 times of magnification, can help in determining fine edges and examining the position and morphology of the edges, inspecting the position of the restoration, and removing excessive adhesives.^43^

Meanwhile, microscope had better illumination power. The head loupe or bench-top microscope provides a coaxial light source when zooming in the field of view. Compared to the arm light of the dental chair, the illumination of the microscope can eliminate all the blind angles in the field of view and avoid the shadow problem caused by the inconsistency in the direction of light source and doctor’s line of sight.^44^

Good ergonomics

The oral microscope provided a good field of view and allowed the dentist to perform oral operations in a relaxed position, preventing musculoskeletal diseases caused by oral treatment.^45^

Better implementation of accurate tooth preparation surgery

After designing of sufficient tooth preparations, the tooth preparation program should be implemented. Currently, the deep-controlling-hole precise tooth preparation method has been developed. The accuracy of tooth preparation volume can be controlled to 0.1 mm by the guidance of TRS guide plate.^46^ Only the magnified field of view under the microscope can assist the accurate implementation of tooth preparation surgery.^47^

Traditional naked-eye preparation technology lacked one-to-one accurate quantitative relationship between the preoperative design, intra-operative preparation and restorations and accurate and rapid measurement. It is difficult to distinguish the amount of present tooth preparation and prepared teeth.^48^

Because the microscopic field of view is small, the depth of field remained shallow, and the space visual reference frame remained narrow. While the naked eye preparation surgery is based on simple visual inspection, the reference frame remains close to the myopia object in daily life, thus causing the surgeon to have familiar space under the naked eye. The amount of experience is not easy to judge the space size and the amount of preparation under the microscope. It is necessary to develop a special tooth preparation technique under the microscope. The most important point is that there must be a measurable three-dimensional (3D) reference system under the microscopic field of view, and so the use of TRS guide with reference frame and high precision reserve quantity relationship during microscopic minimally invasive preparation is recommended.

The restored tooth needs 0.1–0.2 mm resolution at the boundary of the shoulder, soft and hard tissue and normal abnormal tissue, the mating surface, the finish line, etc. Clearing the adhesive glue even requires a smaller resolution. The need for examination of tooth, pulp, periodontal health, and restoration quality is not attained by the naked eye preparation technique itself.

Therefore, esthetics are strongly recommended for the repair of tooth preparation using a more precise micro-invasive dental preparation technique.

#### Application of oral magnifying device is the visual basis for minimally invasive tooth preparation

A clear view is essential for performing dental treatments, especially for the minimally invasive dentistry, as the boundary between the healthy and the carious tissue is 0.3 mm margin,^49^ the enamel–dentin junction, etc. are undistinguishable with naked eyes. In fact, the theoretical visual acuity of the human eye is about 70 μm, but vision occurring in a gaseous environment (air), diffraction, and refraction reduce it to about 150–200 μm. In dentistry, these values can be compromised even further by the low luminosity in the buccal cavity.^50^ Devices of clinic microscopes and head-mounted loop, which equipped dentists with accurate views, are thus considered as visual basis.^51^

Head-mounted loop was first used in clinical treatment by Saemmish in 1876. Afterwards, Baumann used the loop in dental treatments. In 1978, Dr. Apotheker and Dr. Jako investigated a basic form for dental microscopes and invented the first dentiscope for dental surgeries in 1981. However, owing to its ergonomic discomfort and simple configurations, practical use of dentiscope remained difficult and hence limited its use. In 1992, Dr. Gary Carr first introduced an ergonomic dental operating microscope (DOM) for endodontics. It provided magnifying scales beyond human naked eyes and coaxial vision, satisfying the need for root canal therapies (RCTs).^52^ Till now, DOM is not only limited to endodontics but is also used in fields of periodontics, prosthodontics, and oral and cosmetic surgeries.^53^ Microscope usage in dental operations not only offered the advantage of high visual distinguishability but also a healthy operation position, decreasing mental and physical pressure for practitioners, increasing the concentration, and eventually a pleasing restoration work.^54^ The alternatives of magnitude level are useful for adaptation to various dental treatments. During tooth preparation, magnified view increases the accuracy of the preparation and leads to accurate margins and finishing lines. For the try-in and bonding processes, the micron level overhangs and the gaps as well as slight remnant binding materials are distinguishable with magnified views, ensuring for a long-term desired restoration effect. In addition, the comfortable operation position also relieves occupational joint and muscle pains.

Table 1 presents the advantages and disadvantages of head-mounted microscope and DOM. The former is lower in price, comfortable in ergonomic operation posture, and provide medium magnification scale. Disadvantages include: (1) single magnification scale that is unavailable for precise operations requiring high-level magnifying view; (2) operation posture promotion is limited; (3) the weight of the microscope itself could increase the cervical pressure; (4) video anti-shake is difficult and costly.^55–57^ Comparatively, DOMs are more advanced in: (1) alternative scales with high-level magnifications; (2) better ergonomic posture; (3) coaxial illumination device equipped; (4) video recording system with high-performance cost ratio.^50,58,59^

**Table 1:** Comparison of head-mounted microscope and dental operating microscope

Item	Head-mounted microscope	Clinic microscope
Price	Low	High
Magnitude	Low and nonadjustable	Adjustable with wide ranges
Ergonomic	Good	Excellent
Illuminations	Non-coaxial	Coaxial, without shades
Digital assisting devices	None	Capable of mounting many devices

**Table 2:** Pharmacological properties of the regular anesthetics

	Onset time/min	Duration time/min	Maximum dose/(mg·kg^–1^); absolute value/mg	Toxicity	Effect time
2% lidocaine (1:100 000 ratio with epinephrine)	2–3	60 (Pulp) 180–300 (soft tissue)	4.4 mg·kg^–1^; 300 mg	Low	Medium
2% mepivacaine (1:20 000 ratio with norepinephrine)	1.5–2	60 (Pulp) 180–300 (soft tissue)	4.4 mg·kg^–1^; 300 mg	Low	Medium
3% mepivacaine (no epinephrine added)	1.5–2	20–40 (Pulp) 120–180 (soft tissue)	4.4 mg·kg^–1^; 300 mg	Low	Short
4% articaine (1:100 000 ratio with epinephrine)	2–3	75 (Pulp) 180–300 (soft tissue)	7.0 mg·kg^–1^; 500 mg	Low	Medium
0.5% bupivacaine (1:200 000 ratio with epinephrine)	6–10	90–180 (Pulp) 240–540 (soft tissue)	1.3 mg·kg^–1^; 90 mg	High	Long
Tetracaine	20	20–60 (soft tissue)	20 mg	High	Short
Benzocaine	0.5–2	5–15 (soft tissue)	unknown	Low	Short

In the prosthodontics field, DOM selection is different from the endodontics. A few demands should be noted: (1) larger view even in the same magnification; (2) depth of field; and (3) light with appropriate color temperature.^51^

#### Tooth tissue is non-regenerative in clinics and has ethically confirmed conservative principle

Although the tooth tissue has the largest mechanical strength in human tissues, the tooth is also considered as the most fragile tissue due to its non-renewable character.^60^ During prosthodontics treatment, restoration spaces are provided by hard tissue removal of the tooth. Restoration replacements could occur several times in patient’s life due to possible secondary caries or biological and mechanical complications. Further tooth preparation is required for restoration renewal, but the amount of tooth tissue is permanent. Therefore, from the first preparation operation, minimal reduction and preserving of tooth tissues are considered for long-term oral health and medical ethics.

#### Accuracy requirements for tooth preparation

Since the tooth tissue could not be regenerated clinically till now, the accuracy of tooth preparation ranked the highest of all dental treatment procedures. With the continuous advancements and development of dental materials, the thickness required for the strength of the restoration is becoming smaller and smaller, making it possible for the preservation of more tooth tissues. At present, the minimum thickness for restoration is about 0.1–0.3 mm.^5^ Therefore, the accuracy of preparation has been increased to 0.1 mm, which has exceeded the limit of the human eye.

With the guidance of guide plate and actual measurement by periodontal probe, the accuracy of periodontal surgery and implant surgery can be improved to 1 mm.^61^ The bone tissue and soft tissue have certain flexibility. The accuracy of 1 mm was acceptable, but it remains difficult to meet the accuracy requirement of 0.1–0.2 mm that is required for precise tooth preparation in porcelain veneer minimal invasive restoration. The accuracy of microscopic tooth preparation has been increased from the traditional free hand, the fixed deep groove to the silicone rubber guide, and the TRS tooth preparation guide.^62^ The control of accuracy in free hand method is empirically guided and had the lowest precision; the fixed depth of the groove with reference to the original tooth surface and silicone rubber indicator guide plate could not guide completely the tooth preparation that required morphological changes, and it can only be measured not all but at some selected sites by periodontal probe. The actual measurement accuracy is the accuracy of periodontal probe, which can only reach 1 mm, and the requirement of <1 mm can only be visually estimated, and the accuracy requirement of 0.1–0.2 mm cannot be easily achieved. However, the tooth preparation guide plate with the reference of future restoration space selected should contain the smallest space required for the restoration, and the preparation volume remained the smallest. The accuracy can be up to 0.1 mm by the actual measurement with the measuring rod, which met the accurate requirement of porcelain veneer minimal invasive esthetic restoration.

#### Precise analysis designing and guiding techniques are the core of minimal invasive tooth preparation

TRS designing is a blueprint for minimal invasive tooth preparation.^10^ Guidance techniques are chosen for transferring the designs to practical preparation effects. The precision of the guidance techniques dominates the accuracy of the preparation.^63^ The guidances are divided into two categories according to its reference: (1) referencing existing tooth space; (2) referencing TRS.

When driven by the existing tooth contour, an even layer of tooth tissue would be reduced. Relevant guidance techniques include freehand, depth groove, and dimple and depth cutter. Investigations found that the mid-third incisal region on the labial face is underprepared by free hand and also suffers a low accuracy. Meanwhile, dimple and depth cutter method have higher preparation accuracy when compared with freehand and depth groove.^64^ Generally, in cases with thin enamel thickness, referencing to the original tooth contour has high risk of dentin exposure. The technique is considered inappropriate for cases requesting tooth contour promotion.^65^ The scope of its application is small, and it is impossible to simultaneously detect the amount of tooth preparation and accurate preparation.

TRS-driven techniques could conquer the above-mentioned issues. A case-specific wax-up would be fabricated by dentists and technicians preoperatively. Tooth preparation would, therefore, be guided by the wax-up space. Specific methods include silicon index guides, mock-up guides, transparent dental diaphragm guides, and 3D printing guides.

##### Wax-up

According to the esthetic analysis and target restoration expectation, esthetic diagnostic wax-up is a 3D output of digital smile design on the plaster model of the patient, which is physical or digital.^66^ It has many applications, including (1)displaying the contour for target esthetic restorations; (2) molding of resin mock-up and provisional restoration, transferring esthetic designs; (3) molding for silicon index, transferring esthetic designs and tooth preparation guidance; and (4) guiding for gingivoplasty and alveolar osteoplasty. Esthetic wax up is a general guidance for the whole esthetic restoration procedure^67^ and is highly recommended for physical or digital esthetic diagnostic wax-up.

Mock up is preoperationally made temporary resin material under the guidance of silicon template, which is a mould of wax up.^68^ Therefore, the mock up shares identical contour as TRS. Using mock-up referencing, the methods of dimple depth cutter or depth controlling hole were combined, as the tooth would be prepared to provide the exact TRS required. In situations with thin enamel, the mock-up technique first expands the tooth range, thus avoiding the dentin exposure. The method could be applied for contour altering cases as well.^69^

##### Tooth preparation guide/TRS guide

The tooth preparation guide is an auxiliary tool used in the process of tooth preparation process, which is mainly used to quantify and visualize the space between the preparation and the shape of the target restoration. This accurately controls the tooth preparation amount to make the tooth preparation in accordance with the requirements of TRS and porcelain design.^70^ The tooth preparation guide is prepared using silicone rubber or dental transparent film based on the target restoration (wax-up) or is made up of 3D printing resin material by 3D printing technology according to the digital wax-up model. TRS guides can be used not only for tooth preparation but also for spatial analysis, restoration assessment, etc.

The TRS guide plate can be used not only to guide the esthetic design to verify the virtual design results but also can be used to accurately guide the tooth preparation with the combination of the depth calibrated drill, ensuring that the future restoration space coincided with the designed TRS when making the restoration. Therefore, the TRS guide plate run through the entire clinical restoration processes, which effectively reduced the TRS transmission error caused during the processes of duplicating the model and artificially making the wax model, thereby achieving the purpose of accurate restoration. According to the differences in craftsmanship of the guide plate, the TRS guide plate can be divided into TRS guide plate made of silicone rubber, transparent dental diaphragm TRS guide plate, 3D printing TRS guide plate, and others.

(1) TRS guide made of silicone rubber

The traditional guide plate was made up of silicone rubber to duplicate the esthetic diagnostic wax model and obtain the silicone rubber tooth preparation guide.^71,72^ This method is simple, the materials were easy to obtain, and the TRS guide plate technique is the easiest to grasp by clinicians. Meanwhile, the classic guide plate manufacturing method also had various shortcomings: owing to opacity of silicone rubber, it cannot achieve the visualization of TRS, as well as accurate measurement of the distance between the tooth preparation guide plate and the abutment, and also the clinical operation was complicated. In addition, since the silicone rubber still had certain plasticity after hardening, its accuracy cannot be guaranteed, and the silicone rubber guide plate cannot continue to guide the preparation of restoration after tooth preparation.^62^

(2) Transparent dental diaphragm TRS guide plate

Dental transparent diaphragm TRS guide plate is made by duplicating the esthetic diagnostic wax model into a plaster model and then pressing the dental transparent diaphragm.^73^ Compared with the traditional silicone rubber TRS guide plate, the transparent dental diaphragm not only had better visibility, higher strength, and fitness but also can be used in the entire process of restoration. Hence, it is considered an ideal method for making TRS guide plate.

(3) 3D printing TRS guide plate

3D printing (3DP, commonly known as 3D printing), also known as additive manufacturing technology, is a layered manufacturing technique that uses computer-aided design software or reverse engineering reconstruction 3D digital model and then dividing them into layer-cut data files, with layer-by-layer superposition of materials on a 3D printing device according to the data of each layer, and ultimately forming the object entities. In 1990s, the 3D printing technology has been applied in the production of complex models in the medical field.^74^ Currently, the 3D printing technology has been gradually applied in the field of stomatology.^75^ According to the molding method, it mainly included stereolithography (SLA), selective laser melting, fused deposition modeling, and inkjet printing for the production of TRS guide plate, implant guide plate, prosthetic fusible wax model, removable and fixed denture metal stents, maxillofacial prostheses, complete dentures, etc.^76–81^

3D printing TRS guide plate refers to the conversion of esthetic analysis and the designed TRS digital results into entities through 3D printing technology. As an important means of advance notice for esthetic restoration and accurate implementation, it reduced the steps such as model preparation and manual production of esthetic diagnostic wax model, effectively reduced the TRS transmission error caused by duplicating the model and the manual production of wax model, and effectively improved the diagnosis rate and patients’ comfort.

According to the equal thickness of TRS guide plate in 3D printing, it is divided into two types: 3D printing equal-thickness guide plate and 3D printing non-equal-thickness TRS guide plate.^46^

(A) 3D printing equal-thickness TRS guide plate

**Fig. 3:**
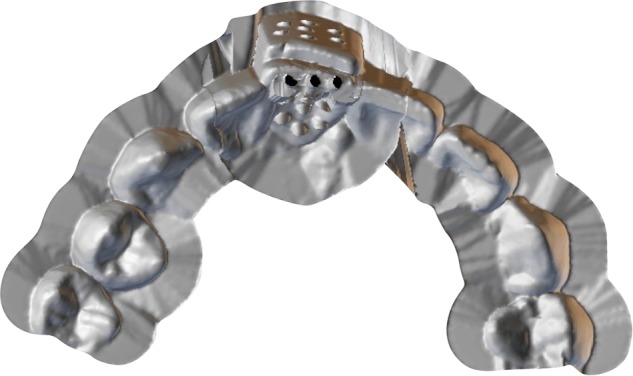
Three-dimensional printing of equal-thickness targeted restorative space guide plate

The equal thickness TRS guide plate made by 3D printing can directly assist in designing the digital guide plate based on the digital esthetic diagnostic wax model and then print the guide plate through SLA technology, which is simple and convenient. This only requires introduction of esthetic diagnostic wax model data that is completed in the stage of analysis and design by professional design software directly by conducting the “shelling” operation based on this data, where the thickness of TRS guide plate is set to a certain target thickness (such as 0.8 mm),^46^ reserve several sites required for tooth preparation to complete the design of tooth preparation guide plate, and finally the TRS guide plate is printed with 3D printing. The guide plates printed by this technology were equal in thickness (Fig. 3). At this time, the inner surface of the guide plate is the outer surface of the future restoration. When using an accurate depth controlling hole in tooth preparation technology, it should be noted that the depth required for keeping in different areas of the TRS should be different.

(B) 3D printing of non-equal-thickness TRS guide plate

**Fig. 4:**
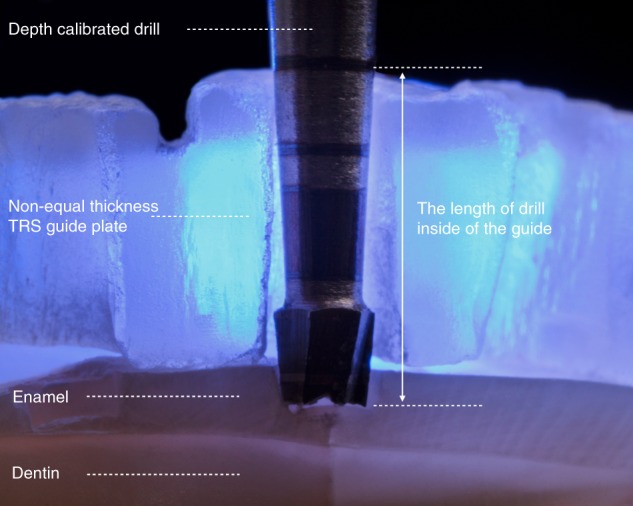
Three-dimensional printing of non-equal thickness targeted restorative space guide plate to ensure the consistency of the depth of the depth calibrated drill into the guide

**Fig. 5:**
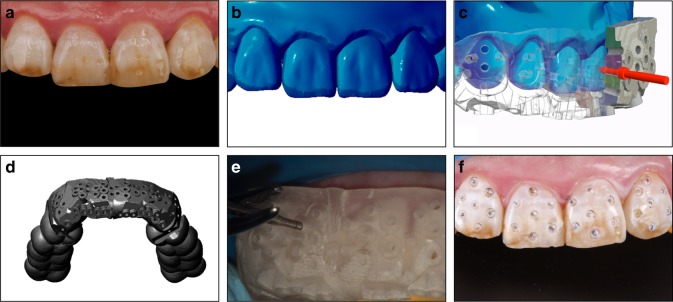
Three-dimensional (3D) printing of non-equal-thickness targeted restorative space (TRS) guide plate tooth preparation. **a** Original teeth. **b** Digital wax-up. **c** Cross-section view of 3D printing of non-equal-thickness TRS guide plate. **d** Overall view of 3D printing of non-equal-thickness TRS guide plate. **e** Preparing depth controlling holes with 3D printing TRS guide plate and depth calibrated drill **f** Depth controlling holes on the teeth surface

To avoid the confusion and inconvenience that may occur in the actual application of 3D printing non-equal-thickness guide plate and to simplify the steps of depth controlling on TRS guide plate using the depth calibrated drill, the 3D printing TRS guide plate can be made into non-equal-thickness guide plate (Fig. 4), where the inner surface of the guide plate is still the outer surface of the future restoration, but the thickness of the guide plate is not equal, so that the depth of the depth calibrated drill into the guide plate remained consistent in each preparation area, and the process of depth controlling preparation is simplified, keeping it more procedural and “automated”. The tooth preparation method can be seen in Fig. 5.

#### Appropriate finishing plane/line design and accurate tooth preparation guarantees long-term stability

The novel concept of finishing plane and cutting plane is to obtain the maximum enamel bonding surface, which in turn affects the long-term and stable bonding performance. Relevant factors include bonding and esthetic (mainly the finishing line).

**Fig. 6:**
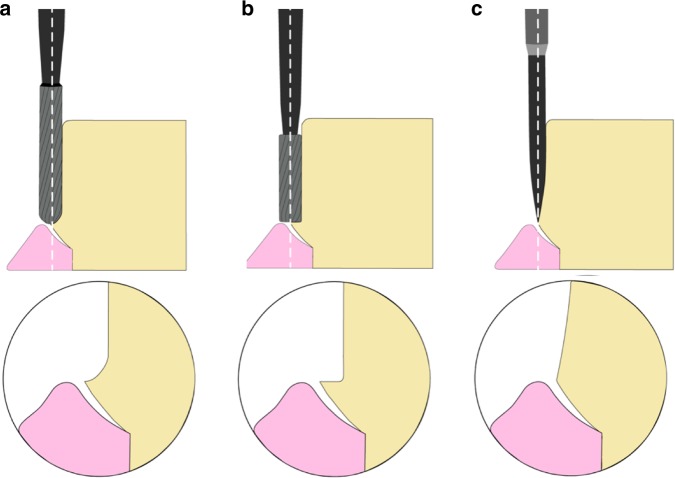
Tooth preparation burs and edge designs. **a** Chamfer edge. **b** Shoulder edge. **c** Feather edge

Marginal design impacts on the periodontal tissue, namely, the red esthetic, are another key point for esthetic minimal invasive design.^82^ The contour and position of the preparation margin are controversial and many factors should be taken into consideration.^83^ Although the developed bonding techniques provided ultra-thin ceramic veneers with sufficient marginal strength, the margin’s preparation, transfer operations, and restoration fabrication are considered as the influential factors. Generally, the margin geometry is divided into three classifications: chamfer edge, feather edge, and non-edge (Fig. 6). Chamfer edge is the most prevailed design and is a “horizontal” design. Clinically, 0.3 mm margin is prepared with a diamond bur or carbide bur. The distinct width assists the technicians to distinguish and fabricate the final restorations. Meanwhile, the thickness of margin allows for mechanical strengthening of the restoration, preventing margin fracture during try-in. Feather edge design belongs to the “vertical” type. Compared with chamfers, feather edges have less preparation amount, ensuring larger enamel bonding surface and bonding strength, and are therefore more conservative. However, in practical cases, the ambiguous finishing line causes difficulty for dental technicians to confirm the precise position.^84^ The thin margin of the feather edges also raised difficulties for both technical fabrication and dentists during operation. During try-in and bonding procedures, the thin margins are more likely to cause fractures. In recent years, some scholars have suggested that vertical edges can be used in the esthetic area, which was completely a non-shoulder preparation. This kind of preparation had no clear termination line but a planar termination area.^85^ Non-prepared margin is used only for ETRS cases. Although the design is completely non-invasive, its performance has similar limitations as that of feather edges and remain difficult for operation. Therefore, the usage of a 0.3–0.5-mm chamfer edge design is recommended.

According to the position comparison with gingival margin, the restoration margin can be classified into three types: supragingival margin, equigingival margin, and subgingival margin.^86^ Since the minimal invasive ceramic veneers are mainly chosen for the anterior teeth, the supragingival margins that influence the esthetic effects are limited to the proximal and lingual areas for teeth without color alteration. The supragingival margins still have the advantages of preparation and are transfer friendly as well as have anti-caries property due to self-cleaning effect.^87^ The controversial subgingival margin could guarantee a natural and esthetic effect for cases with discoloration. At the same time, it is criticized for its complexity in etching and bonding operation and the difficulty for cleaning.^88^ Some clinicians believed that the strict operation procedures lead to a perfect marginal fit, achieving a proper bonding and cleaning effect. For feather edge and non-prepared edge designs, the subgingival designs further increased the difficulty to confirm the marginal position. Equigingival margin is therefore the regular choice for minimal invasive ceramic veneers. The design masks the visual surfaces, shows clear margin position for feather and non-prepared edges, and performs good etching and bonding effects. Accordingly, the designing of finishing plane/line is essential for esthetic restoration, bonding stability, and periodontal health. Therefore, it is strongly recommended to retain more enamel, try to use an equigingival margin or subgingival margin design, and against excessive deep preparation of edge zone.

#### Tooth preparation burs

There are mainly two types of tooth preparation burs depending on the material: diamond burs and carbide burs. The diamond burs have high cutting efficiency and are suitable for massive tooth preparation amounts.^89^ The prepared surfaces are scratched and then are polished. The grains are coated on the stainless steel bur body and could be worn off during cutting, resulting in a lowered cutting efficiency and cutting quality. The smooth carbide burs are capable of preparing precise and polished tooth surfaces, allowing for an accurate impression and marginal fitting ceramic restoration.^90,91^ Owing to its material and shape, the carbide burs are more durable and have stable cutting performances even after several clinical applications. Overall, the carbide burs are most advisable for minimal invasive tooth preparations, especially suitable for the preparation of high-quality finish line and shoulders.

To guarantee a precise tooth preparation amount, the selection of bur instrument selection remains to be an influential factor. On different tooth sections, the preparation details varied and the corresponding bur types also changed. The round burs could be used for preparing labial and lingual preparations; calibrated depth cutter burs could mark the preparation depth for the labial and buccal surfaces; coned burs are used for tooth tissue removal and finishing line preparation and specific tip designs coincide with the margin designs; and the spindly burs are used for proximal interface cutting. As the shape of the bur tip determines the shape of the shoulder and the finishing line (Fig. 6), it is strongly recommended to select tungsten burs with tips of corresponding shape and size to improve the margin quality. However, when the wrong diameter of burs is selected, there is the flash at the margin.

#### Polishing

Previous reports showed that increased abutment tooth roughness benefited restoration retention. However, overt abutment tooth roughness leads to difficulties in final impression and causes irregular small bulges and high interferences that impact the insertion and fit of restoration.^92^ With the converge of abutment tooth forms, the gaps caused by the rough surface of irregular bulges would enlarge, resulting in lowering of retention and occurrence of microleakage.^93^ Therefore, polishing of the abutment tooth aims to smooth out the irregular interferences and increase the fitness with final restoration. Moreover, the smear layer could be removed by polishing and thus increases the surface activity.^94^ Polishing procedure is a sequential process that involves rough to fine polishers.^95^ In dental cases, the interference points would be polished successively with diamond burs, carbide burs, rubber polisher, lint polisher, and other instruments to obtain a smooth abutment surface. The smoothened surface to some extent would decrease the bonding strength.^96^ The issue could be solved by excellent mechanical properties of novel resin-bonding systems and the pre-bonding sand blasting, roughening the abutment surface. The physical optimization after polishing could compromise the decreased retention as well. Consequently, the polishing of abutment tooth is overall beneficial.

#### Immediate dentin sealing (IDS), promotion for the immediate and long-term effect

Conventionally, the bonding agents would be applied during the final try-in of restoration. In 1996, Bertschinger^97^ found that bonding agent treatment immediately after tooth preparation illustrated better immediate and long-term bonding strength, and hence the concept of IDS has been put forward. Specifically, IDS refers to the bonding agent treatment on exposed dentin tissue immediately after preparation.^98^ The process is as follows: (1) distinguish the dentin exposure regions; (2) etching the target region for 5–15 s; (3) rinse and dry; (4) apply bonding agent, blow to a thin layer; (5) preliminary light curing; and (6) application of antioxidant and further light curing. IDS is necessary for minimally invasive tooth preparation and directly alleviates dentin sensitivity and optimize the bonding effects. Treating enamel and dentin tissue with separate bonding methods also indirectly enhances the final bonding strength.

#### Rubber dam

Rubber dam was first introduced in the microscopic RCT and demonstrated its efficacy and quality.^99^ For microscopic prosthodontics, rubber dam plays an important role as well. By isolating the oral mucosa along with the operation regions, the rubber dam avoids chemical stimulation (for example: etching agent) on the oral soft tissue. In addition, the rubber dam remains effective for preventing the patients from swallowing and inhaling small instruments, fluid, and material filings; blocking patients’ breath from odontoscopes and providing clear view; and decreasing the infection risk.^100^ Among the microscopic prosthondontics, the bonding procedure benefits the most from rubber dam. In cases of esthetic regions, fixing rubber dams on premolars is recommended for good retention. Rubber dam installing techniques include bow technique, wing technique, rubber first application, and clamp first application. Dentists could choose according to the tooth site and specific purpose.^99^ Microscopic prosthondontics have differential demands along with RCT, and therefore unique assistance methods, such as the use of dental floss, Teflon tape, etc, were introduced. Rubber dam with assisting instruments are necessary for minimal invasive tooth preparation.

#### Pain management in microscopic restoration

In the field of prosthodontics, microscopic prosthodontics is considered to be a more accurate and minimally invasive restoration method, which is mostly used for esthetic restoration of vital pulp and often involved in many dental restorations and multi-step sequence follow-ups. At the same time, the microscopic operation is generally time-consuming, and patients show a stronger desire for psychological satisfaction and painless dentistry.^101^ Hence, pain management is particularly important in the clinical treatment of microscopic esthetic prosthodontics, but it has long been ignored in the clinics.^102^ Pain can be controlled using anesthesia from the level of anesthesia technology, while it can only be relieved through psychological counseling and sedation techniques from the level of psychology to optimize the patient’s subjective experience. The painless microscopic prosthodontics using local anesthesia is not only an inevitable requirement for the development of high-precision medical technology but also a medical virtue based on human care. In addition, the patients undergoing esthetic restoration are mostly young people, with a large pulp chamber and high pulp horn, and present more obvious pulp reaction stimulated by temperature variation and dentine sensitivity caused by the exposed dentinal tubules.^103^ Therefore, corresponding measures should be taken for achieving painless effect during and after tooth preparation.

##### Principle for local anesthesia

Requirement of patient consent and physical and psychological evaluations:

Physical condition: the relative contraindications include recent myocardial infarction and cardiac angina, blood coagulation disorders, and infections of the injection area.^104^ For patients suffering from high blood pressure or hyperthyroidism, adrenaline is forbidden. Patients with anesthesia allergy history require sensitivity test.

Psychological condition: evaluate anxiety and fear emotion level to decide whether sedative drugs are needed.^105,106^

##### Local anesthetics

Regular dental local anesthetics include esters or amides. Tetracaine and benzocaine esters are used for surface anesthesia. Lidocaine and articaine amides are used for infiltration anesthesia or nerve block anesthesia. Pharmacological properties of the regular anesthetics are listed below (Table 2).^107^

For an identical site, operations within 1 h allows for a free choice among lidocaine, mepivacaine, or articaine. Bupivacaine or levobupivacaine are more appropriate for long-time-taking operations, as it could avoid repeated injections of anesthesia.^108^ Patients with epinephrine restriction could be treated with simplex mepivacaine.^109^

##### Local anesthesia applications

In microscopic prosthodontics, surface anesthesia and local infiltration anesthesia/nerve blocking anesthesia are often chosen. Local anesthesia is operation-friendly and safe, which is the priority for esthetic cases, where the cancellous bone sites have well infiltration of the drug. For anesthesia of 1–2 tooth sites, infiltration method could be selected. For treatments with >3 teeth or with blood coagulation disorders, nerve-blocking anesthesia could be used to ensure longer anesthesia period and decrease injection frequency.^110^ Superior alveolar nerves or infraorbital nerves are blocked during anesthesia of the maxillary anterior teeth sites. Incisor nerves are blocked for anesthesia in the mandibular anterior teeth sites. In addition, adequate surface anesthesia (>2 min) and slow injection rate (≤1 ml/min) are more comfortable for patients.^111^

##### Relieving postoperative hypersensitivity

The discomfort after tooth preparation mainly comes from the pulp reaction caused by the temperature and other stimulations during the tooth preparation, long-term opening, and body limitation, as well as dentine sensitivity caused by the exposure of the dentinal tubules after tooth preparation.^112^ According to the operating principle of minimally invasive restoration, the range of tooth preparation should be controlled within the enamel as much as possible. Nevertheless, the dentine exposure can be caused by some other reasons, such as excessive protrusion of the tooth. After the anesthetic effect, most of the patients with vital pulp had dentin sensitivity. Before the patients feel uncomfortable, the dentine tubule should be closed to isolate the pulp from external stimuli, thereby avoiding secondary damage caused by stimuli. The dentin sensitivity can be reduced or eliminated by two ways. One is by reducing the local nerve sensitivity using drugs, and the other is by blocking and obstructing the dentin tubules. The drugs that reduce local nerve sensitivity mainly include potassium salts such as potassium citrate and potassium nitrate.^113^ In addition, immediately after the tooth preparation and before preparing the final impression, application of dentin-bonding agent showed good permeability to seal the dentinal tubules, which enhances the adhesion to protect the compound of pulp and dentin and also prevents the dentin sensitivity during the wearing of temporary restoration.^114^

## Conclusions

Minimal invasive tooth preparation assists for long-term stability of esthetic restorations. Several principles should be noticed by prosthodontic dentists:

1. It is suggested that psychological, physiological, functional, and esthetic factors should be considered comprehensively to analyze the TRS before the treatment, and appropriate restoration methods and materials should be selected. The bad psychology and functional decompensation are considered as contraindications for esthetic restoration. What’s more, uncontrolled periodontal diseases, dental pulp diseases as well as severe wear and TRS insufficiency etc., should also be considered as relative contraindications.
2. As the tooth tissue is limited, it is strongly recommended to preserve the tooth and protect the vital pulp for the prepared tooth, master the anatomical relationship and thickness of different parts of enamel at different positions, and make preparations that conform with the anatomy and physiology of the teeth. The thickness of the remaining dentin should also be paid attention to avoid irritation of the pulp and maintain the health of the pulp. The complete surface should be designed as much as possible in the enamel layer to achieve a stable adhesion repair effect.
3. It is strongly recommended to use microscopic equipment to ensure accuracy of 0.1 mm and effect the tooth preparation, as well as the ergonomic convenience of the operators.
4. It is highly recommended to prepare diagnostic wax models for determining the TRS and use the appropriate preparation guide technology to quantify accurate tooth preparation under the guidance of high-precision TRS guides.
5. The designs should be reasonably designed according to the width and shape of the shoulder to reduce the damage of the tooth cervix and periodontal tissues. It is recommended to use supragingival and equigingival margins, the 0.3–0.5 mm mini-chamfer, non-360° shoulders, etc. to ensure the health of the tooth cervix and periodontal tissues.
6. It is recommended to use tungsten steel bur with the tip shape and size corresponding to the design to improve the quality of the finish line preparation. It is also recommended to use a high-quality cutting system to reduce iatrogenic damage to the dental pulp and periodontium.
7. It is recommended to use IDS, desensitization, and other techniques to reduce the complications, such as increased postoperative sensitivity.
8. It is recommended to adopt whole-course painless technique.

## Supplementary information

Supplemental Material File #1

Title page
