# Supplementary figures and images for: Minimal invasive microscopic tooth preparation in esthetic restoration: a specialist consensus
Source: Int J Oral Sci. 2019 Oct 2;11(3):31. doi: 10.1038/s41368-019-0057-y (PMC6802612; doi:10.1038/s41368-019-0057-y)

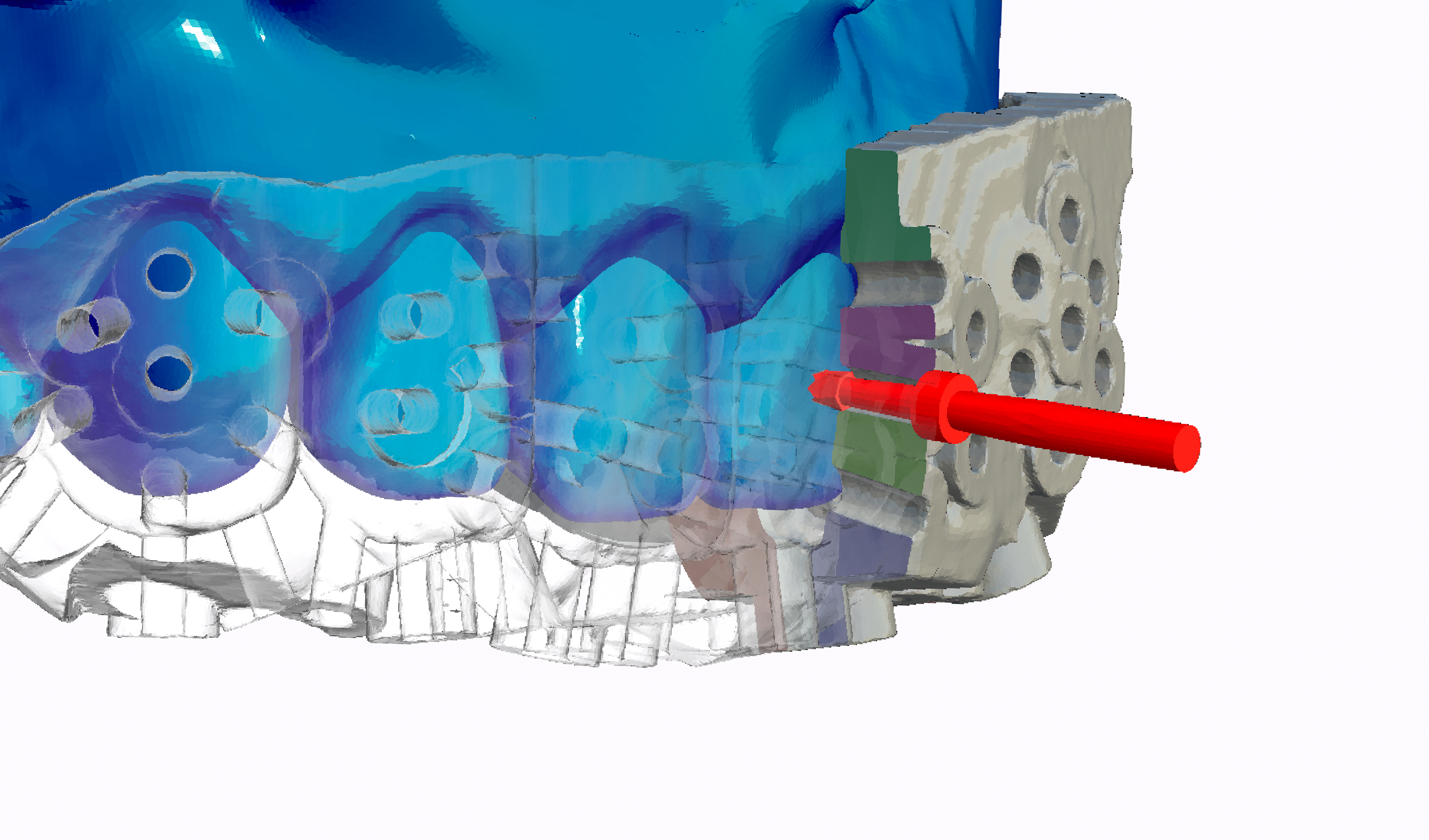

Supplement: Supplementary file 1 — Supplemental Material File #1 [file 41368_2019_57_MOESM1_ESM.png]
