# Supplementary material for: Minimal invasive microscopic tooth preparation in esthetic restoration: a specialist consensus
Source: Int J Oral Sci. 2019 Oct 2;11(3):31. doi: 10.1038/s41368-019-0057-y (PMC6802612; doi:10.1038/s41368-019-0057-y)
Supplement: Supplementary file 2 — Title page [file 41368_2019_57_MOESM2_ESM.docx]

Minimal invasive Microscopic Tooth preparation in esthetic restoration: a specialist consensus.

Haiyang Yu^1,*^, Yuwei Zhao^1^, Junying Li^1^,Tian Luo^1^, Jing Gao^1^ , Hongchen Liu^2^,Weicai Liu^3^, Feng Liu^4^, Ke Zhao^5^, Fei Liu^6^，Chufan Ma^7^, Juergen M. Setz ^8^, Shanshan Liang^9^, Lin Fan^1^, Shanshan Gao^1^, Zhuoli Zhu^1^, Jiefei Shen^1^, Jian Wang^1^, Zhimin Zhu^1^, Xuedong Zhou^1,*^

1. State Key Laboratory of Oral Diseases, National Clinical Research Center for Oral Diseases, West China Hospital of Stomatology, Sichuan University, China, 610041

2.Chinese PLA General Hospital, Chinese PLA Medical Academy, China, 100036

3. Department of Stomatology Digitization, Hospital of Stomatology, Tongji University, China, 200072

4. Department of Prosthodontics, Hospital of Stomatology, Peking University, China, 100871

5. Department of prosthodontics, Guanghua Stomatological Hospital, Sun Yat-sen University, China, 510055

6. Department of Biologic and Materials Sciences and Division of Prosthodontics, University of Michigan School of Dentistry, US, MI 48109

7. Department of Prosthodontics, School of Stomatology, The Fourth Military Medical University, China, 710032

8. Department of Prosthodontics, Hospital of Stomatology, Martin-Luther-University, Halle (Saale), Germany, 06097

9. Department of Prosthodontics, Hospital of Stomatology, Wuhan University, China, 430079

Haiyang Yu^1,*^,yhyang6812@foxmail.com (corresponding author)

Yuwei Zhao^1^,yuweidentist@foxmail.com

Junying Li^1^, chqjmd@qq.com

Tian Luo^1^, 289538292@qq.com

Jing Gao^1^ , [jing182025@foxmail.com](mailto:jing182025@foxmail.com)

Hongchen Liu^2^, Liu-hc@301dent.com

Weicai Liu^3^, weicai_liu@tongji.edu.cn

Feng Liu^4^, dentistliufeng@126.com

Ke Zhao^5^, dr.zhaoke@aliyun.com

Fei Liu^6^, feiliu@umich.edu

Chufan Ma^7^, machufan@fmmu.edu.cn

Juergen M. Setz ^8^, juergen.setz@uk-halle.de

Shanshan Liang^9^,liangshanshan@whu.edu.cn

Lin Fan^1^,783792515@qq.com

Shanshan Gao^1^, christina12357@163.com

Zhuoli Zhu^1^, zzl7507@126.com

Jiefei Shen^1^, shenjiefei@scu.edu.cn

Jian Wang^1^, [fero@scu.edu.cn](mailto:fero@scu.edu.cn)

Zhimin Zhu^1^, [zzhimin@163.com](mailto:zzhimin@163.com)

Xuedong Zhou^1,*^, [zhouxd@scu.edu.cn](mailto:zhouxd@scu.edu.cn) (corresponding author)

Conference: The 17th Dental Show West China, Chinese Stomatological Association Prosthodontics Committee, Chengdu, China, April 27,2018
